# Wild and native plants and mushrooms sold in the open-air markets of south-eastern Poland

**DOI:** 10.1186/s13002-016-0117-8

**Published:** 2016-10-07

**Authors:** Renata Kasper-Pakosz, Marcin Pietras, Łukasz Łuczaj

**Affiliations:** 1 Doctoral Studies Programme, Faculty of Biology and Agriculture, University of Rzeszów, ul. Ćwiklińskiej 2, 35-601 Rzeszów, Poland; 2 Department of Plant Taxonomy and Nature Conservation, University of Gdańsk, Wita Stwosza 59, 80-308 Gdańsk, Poland; 3 Institute of Dendrology, Polish Academy of Sciences, ul Parkowa 5, 62-035 Kórnik, Poland; 4 Department of Botany, Institute of Applied Biotechnology and Basic Sciences, University of Rzeszów, Werynia 502, 36-100 Kolbuszowa, Poland

**Keywords:** Ethnobotany, Ethnomycology, DNA bar-coding, Protected plants, Medicinal plants, Conservation, Fungi

## Abstract

**Background:**

The study of plants and fungi sold in open-air markets is an important part of ethnobotanical enquiry. Only few such studies were carried out in Europe.

**Methods:**

Four of the largest open-air markets of south-eastern Poland were visited regularly, and the plants sold in them were recorded between 2013 and 2015. The aim of the study was to record native and/or wild species sold in the markets. All the plants sold in the markets were photographed regularly. In each market, 25 sellers were interviewed. Voucher specimens were collected and fungi were identified using DNA barcoding.

**Results:**

Altogether, 468 species of plants were recorded, 117 of them native to south-eastern Poland – 19 only collected from the wild and 11 both wild and cultivated. Seventeen of the species are under legal protection. Most protected plants were sold from cultivation, although proper authorization procedures had not been performed. Thirty-two species of fungi were sold (including two cultivated species), all of them for culinary purposes. Two species (*Lactarius quieticolor*, *Leccinum schistophilum*) are new to the mycobiota of Poland.

Ornamental plants constituted a large section of the market, and they dominated the group of native species. Food plants dominated among wild-collected plants and were sold mainly as fruits for jams, juices and alcoholic drinks, or as culinary herbs. Very few medicinal or green vegetable plants were sold. An interesting feature of the markets was the sale of *Ledum palustre* as an insect repellent.

**Conclusions:**

Finding two species of fungi which are new to Poland highlights the importance of DNA barcoding in ethnomycological studies. Most items in the markets are ornamental plants, or edible fruits and mushrooms. Very few medicinal plants and green vegetables are sold, which differentiates the markets from southern European ones. Such a pattern is probably the model for most central European markets.

## Background

The study of plants sold in open-air markets is an important part of ethnobotanical enquiry [1, 2]. Plants which are sold in such places are usually those which are culturally the most salient. In traditional agricultural societies the market is often the main source of goods sold and bought by villagers. Even with the advent of regular shops and supermarkets in more modernized societies, open-air markets remain an important centre of plant commerce for both urban and rural dwellers. Many of the plants sold in the markets come from the wild, thus these places are inherently connected with the issue of sustainable collection of plant material from wild growing populations.

Rich traditions of the sale of plants in markets still persist in Europe, in spite of its high level of modernization. Probably the first regular studies of the ethnobotany of markets in the world, or at least in Europe, were performed by Polish scholars. In 1927 Muszyński [3] made a list of medicinal plants sold in the market of Vilnius (then Poland, nowadays Lithuania). Very soon after, in 1933, Jerzy Wojciech Szulczewski, a local biologist and ethnographer, issued a paper containing a detailed list of medicinal plants, and another about edible and medicinal fungi [4, 5] (later reprinted in [6]) sold in the markets of Poznań, the largest city of western Poland. Just a few years later Szulczewski recorded 56 species of edible and medicinal fungi and 79 species of medicinal plants sold in these markets. His study was re-visited in 2013 and a dramatic reduction in the sales of medicinal plants was observed [7].

Karousou et al. [8] studied medicinal herbs sold on 15 stalls scattered through markets in the three largest cities in Cyprus. A total of 57 taxa were recorded, of which 32 were cultivated and 14 wild. Similarly, Hanlidou et al. [9] studied medicinal plants in Thessaloniki, Greece. The majority (131) of the 172 recorded taxa were of local origin.

Łuczaj et al. [10] studied wild edible greens sold at 11 town markets in Dalmatia (on the southern coast of Croatia). According to the authors, the use of wild green vegetables (leaves, buds, stems) is very widespread in the Mediterranean. In total, 37 species were recorded.

Probably the longest list of plants sold in local markets was recorded by Ertug [11], in the Bodrum area of Turkey, who recorded 390 species on sale. Most of the recorded plants were wild edible plants, although plants used for fodder, medicine or crafts were also noted. Another study which recorded wild food plants in Turkey was carried out by Dogan et al. [12] who surveyed 18 markets in Izmir and found that 46 species of wild edible plants were sold. Nedelcheva and Dogan studied open-air markets on both sides of the Bulgarian-Turkish border. They found that predominantly medicinal plants are sold in Bulgarian markets, whereas in Turkish markets there are many more wild vegetables sold [13, 14].

A number of scholars have researched plants sold in Asian markets. For instance Pemberton et al. [15] surveyed the three largest markets of wild edible and medicinal plants in Seoul, South Korea. Xu et al. [16] and X [17] looked at plants sold in Xishuangbanna, in the tropical part of Yunnan, China. Shirai & Rambo [18] presented the results of research on wild species sold on the main town square in Khon Kaen, in north - eastern Thailand. The diversity of wild species sold there is high; much higher in the rainy season than in the dry season. They found 60 wild species, of which 54 were plants, and 6 mushrooms. Konsam et al. [19] found a large diversity of wild vegetables sold in the markets of Manipur, India. The ethnobotany of open-air markets was also studied in Pakistan, Iraq, Iran and Kyrgyzstan [20–23].

One of the earliest ethnobotanical works on markets is by Bye [1] who recorded medicinal plants sold in three cities in northern Mexico. Several other authors researched the ethnobotany of markets in South and Central America [24]. For example De Albuquerque et al. [25] compared lists of plants sold in the North-East of Brazil in the city of Recife, the capital of Pernambuco state. Between 1995 and 2002 the number of plants sold increased from 58 to 136 species. Many of the plants are used for medicine, but they often have magical or hygienic connotations. A similar dominance of medicinal plants is found in the markets of Bolivia [26].

African markets are also dominated by medicinal plants (see e.g. [27–30]). Ouarghidi et al. [31] recorded medicinal plants sold in the three markets of Marrakech. They found that many of the species were falsifications and actually, false cheaper species are sold under different names. The fact that many important medicinal roots are not readily available in the markets of Marrakech suggests that these wild species may be in danger of extinction, and the scarcity and high demand for some species has led to their replacement by other taxa. The article lists species that are sold as replacements or forgeries. Similar results were obtained by Kool et al. [32] who found several rare and endangered species in the markets of southern Morocco.

In practically all of the above-mentioned studies from around the world the number of wild species sold is higher than the number of cultivated plants. Some authors raise the question to what extent such commerce endangers wild populations [33]. This is, for example, the case in Morocco concerning medicinal plants. The trade of ornamental plants, for example orchids, may also endanger local populations [34].

Another issue is the sale of mushrooms in open-air markets. It is widespread in many countries of the globe and often regulated [35–37], but the taxa which are sold are often not properly documented, due to the lack of voucher specimens. Recent advances in DNA barcoding techniques for fungi enable a more accurate identification of the species [38, 39].

As previously mentioned, the issue of plant commodification is also connected with conservation status – plants are often protected because they are attractive, useful and prone to extinction. Although the first plant protected by law in Poland was the yew-tree (in 1420 by king Władysław Jagiełło – [40]), nowadays a large proportion of protected plants are those which have attractive flowers which make them prone to being picked or dug out for private gardens or for sale [41], for example *Galanthus nivalis*, *Leucojum vernum*, *Orchidaceae*, *Lilium martagon* etc. Such flowers formed the core of protected plants in the first post-World War II law on plant protection in Poland [42]. In Poland the overharvesting of wild medicinal plants used to be a problem, and rare medicinal plants constituted another large sector of protected plants. They were usually partially protected so that the authorities could license their gathering. Some of the species were not very rare at all (e.g. *Asarum europaeum*, *Viburnum opulus*, *Frangula alnus*) and their protection was cancelled in the most recent plant protection legislation [41], as the gathering of medicinal plants is much less widespread now. However, much earlier, at the end of the 19th century, many populations of highland alpine plants in the Tatras were decimated by pickers supplying medicinal “roots” to herbalists [43].

The aim of this study was to record wild plants and fungi sold in the markets of south-eastern Poland. Our hypotheses were:

1. The main wild products sold in the markets are ornamentals, wild fruits and edible fungi. Wild vegetables are not sold in the markets. This is the pattern of consumption of wild foods in Poland, and we expected it to be reflected by the choice of plants in the markets.
2. Some protected and rare wild plants are sold.
3. Few medicinal plants are sold due to the general decrease in gathering activities.

## Methods

The research was performed following the code of ethics of the American Anthropological Association [44] and the International Society of Ethnobiology Code of Ethics [45]. Oral prior informed consent was acquired.

**Fig. 1:**
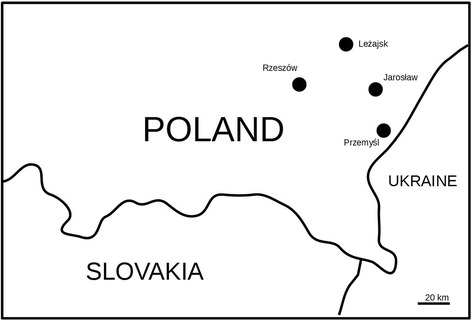
Studied markets

Four open-air markets from southeastern Poland were selected for the study (Fig. 1). This included probably the largest market in this part of Poland, in Rzeszów (190,000 inhabitants), the capital of Podkarpacie region. The other three markets were located in three (out of 21) county towns in the region – Jarosław (ca. 39,000 inhabitants), Leżajsk (14,000) and Przemyśl (63,000).

Open-air markets are located in most towns in Poland. In the past (e.g. before World War II) they were placed in a central market square (a typical feature of Polish medieval towns), but nowadays they were gradually re-located to less central locations. Once the centers of all commerce in towns, including selling animals, now they are a mixture of stalls with cheap clothes, tools, agricultural products, plants and mushrooms.

Data were collected in two ways. The first approach consisted of regular observations of markets. The markets of Rzeszów, Jarosław and Leżajsk (Fig. 1) were visited every week on the days when most people come to buy products (J and L on Tuesday and R on Saturday) in the periods of September-October 2013, and from the end of February to the end of October 2014 and 2015. In 2015, the markets were visited only once every two weeks. Additionally the market in Przemyśl was visited seven times in 2015, from April to October, at monthly intervals. Altogether 13,488 photos were taken. Photographic documentation enabled the quick recording of plants used in public spaces and detailed identification of most taxa at least to genus level [46].

**Fig. 2:**
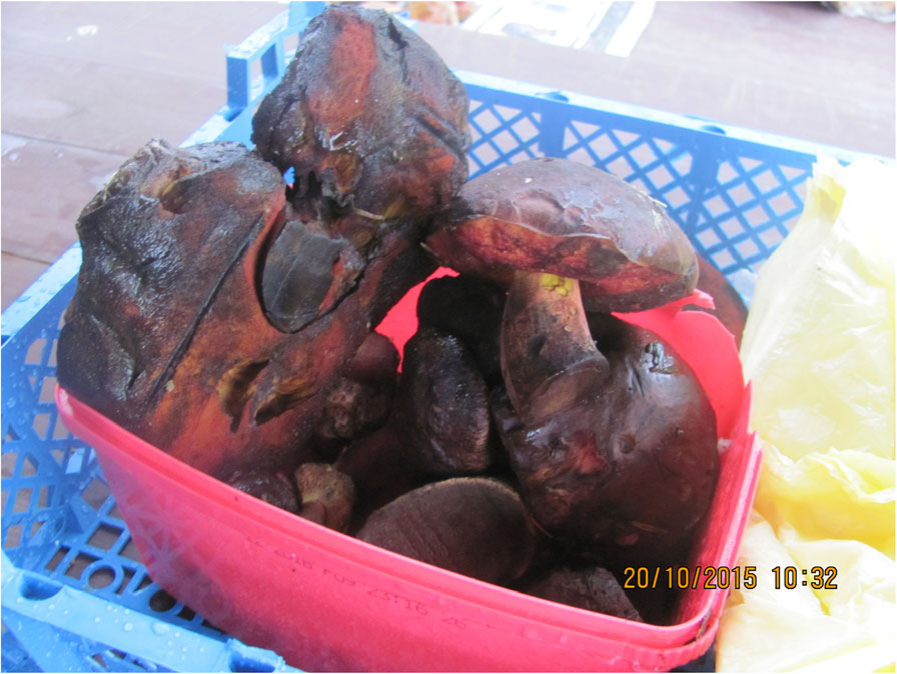
*Boletus luridiformis* is widely sold in SE Poland, though its sale is not permitted in open-air market places (photo from Przemyśl)

**Fig. 3:**
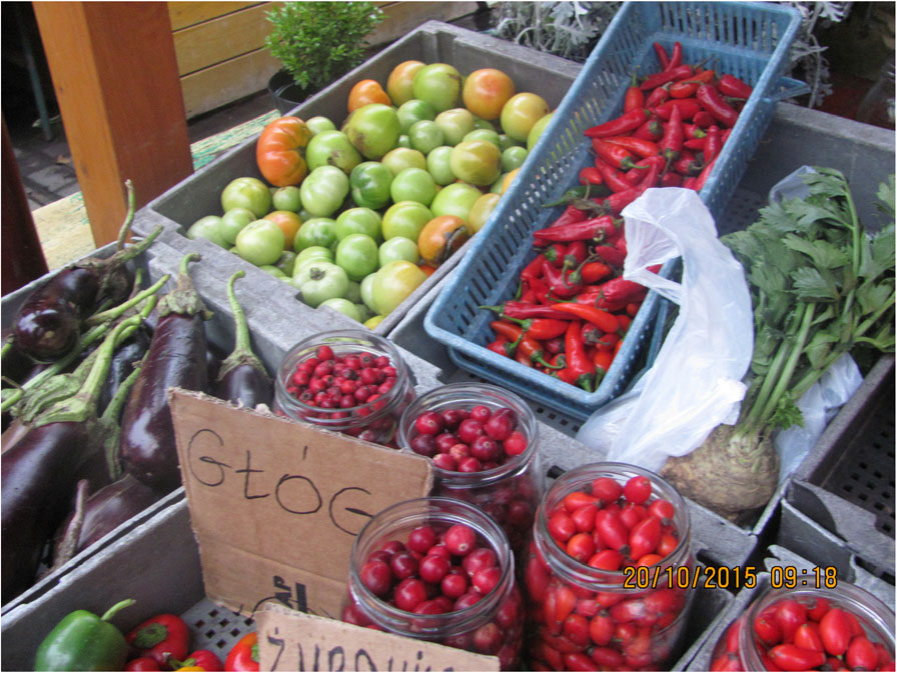
*Rosa*, *Crataegus* and *Oxycoccus* fruits on sale in Jarosław

**Fig. 4:**
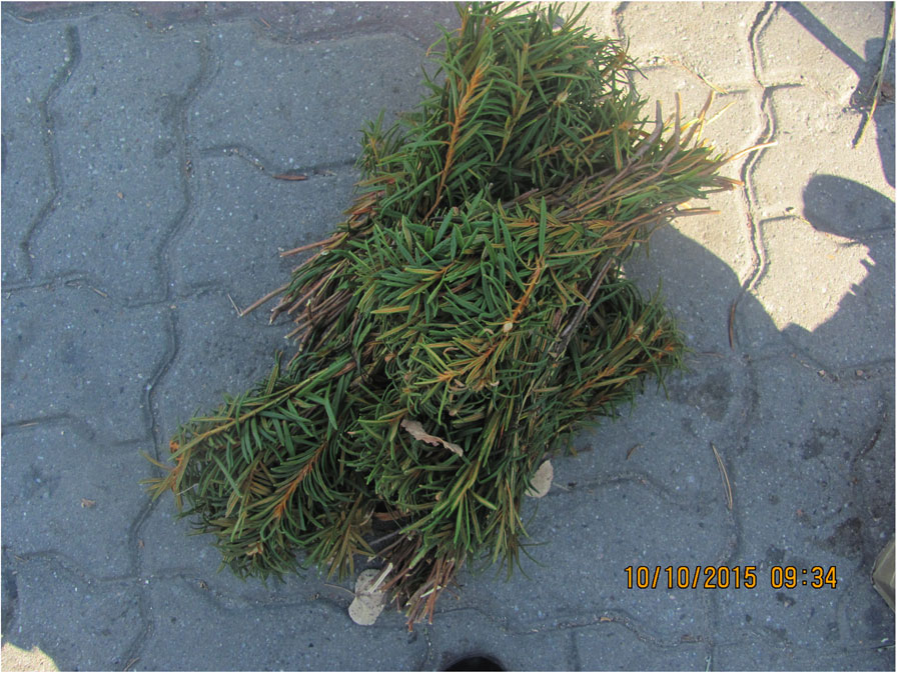
*Ledum palustre* is commonly sold as insect repellent in Rzeszów, in spite of being protected by law

**Fig. 5:**
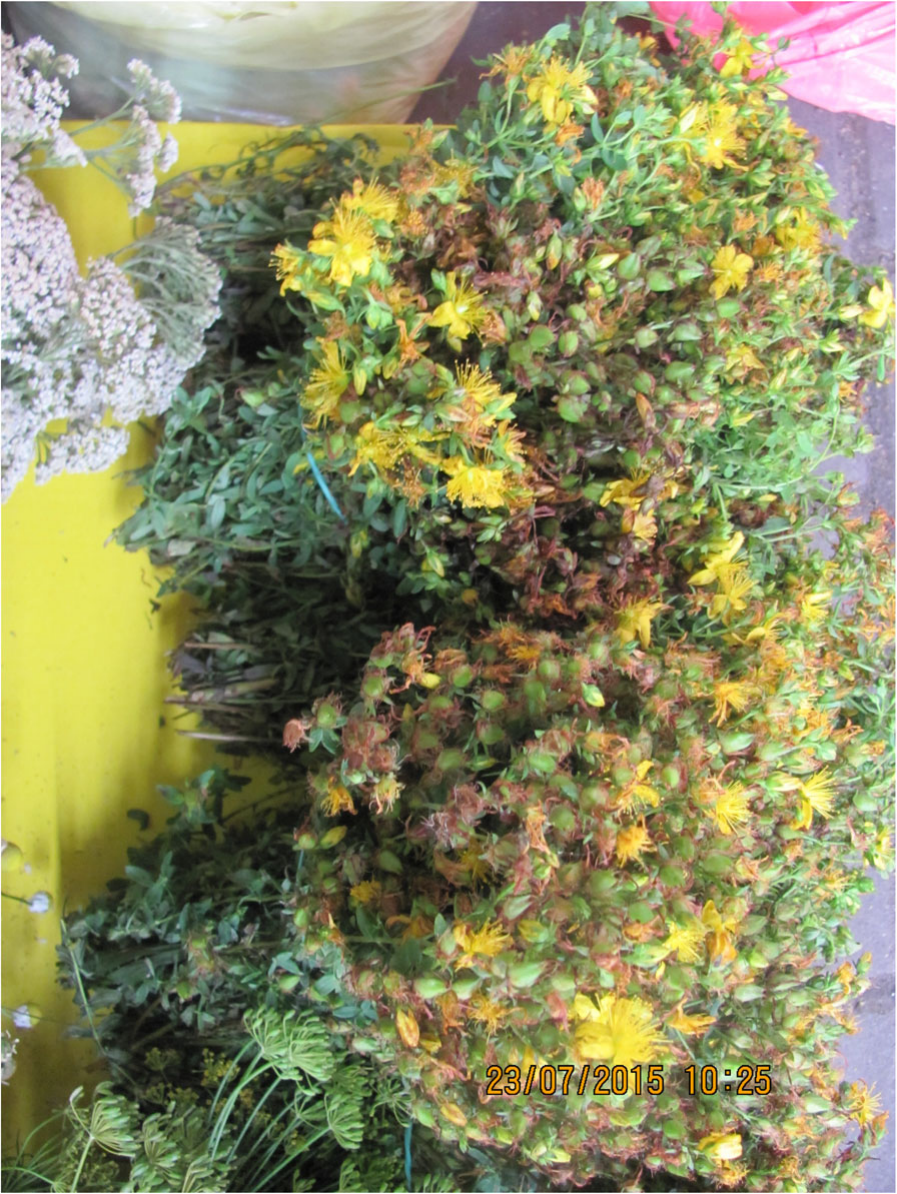
*Hypericum perforatum* is one of the very few purely medicinal plants sold in open-air markets in Poland

**Fig. 6:**
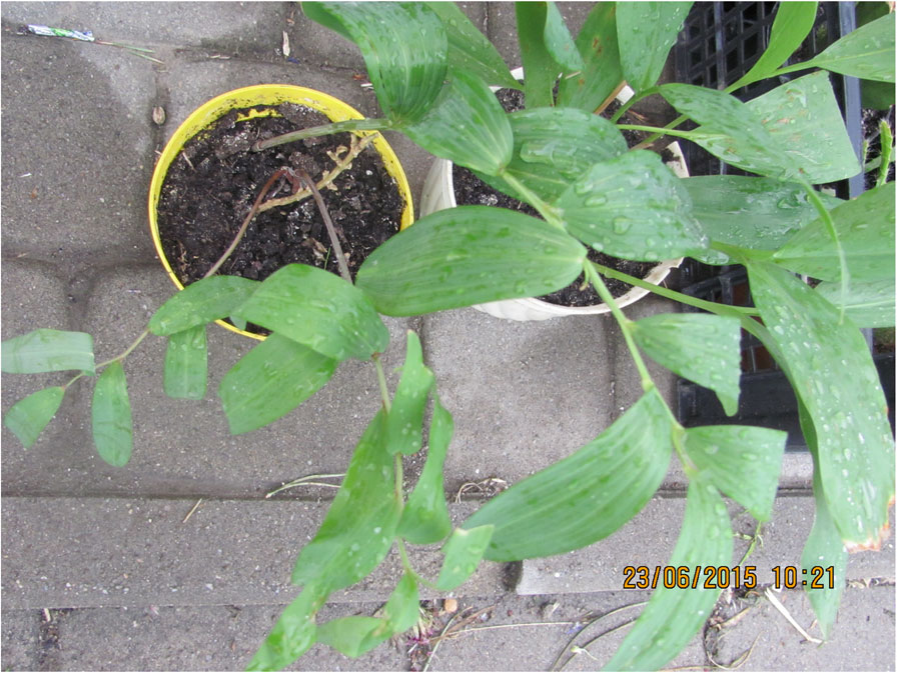
Wild-origin *Polygonatum multiflorum* sold as garden ornamental in Przemyśl

**Fig. 7:**
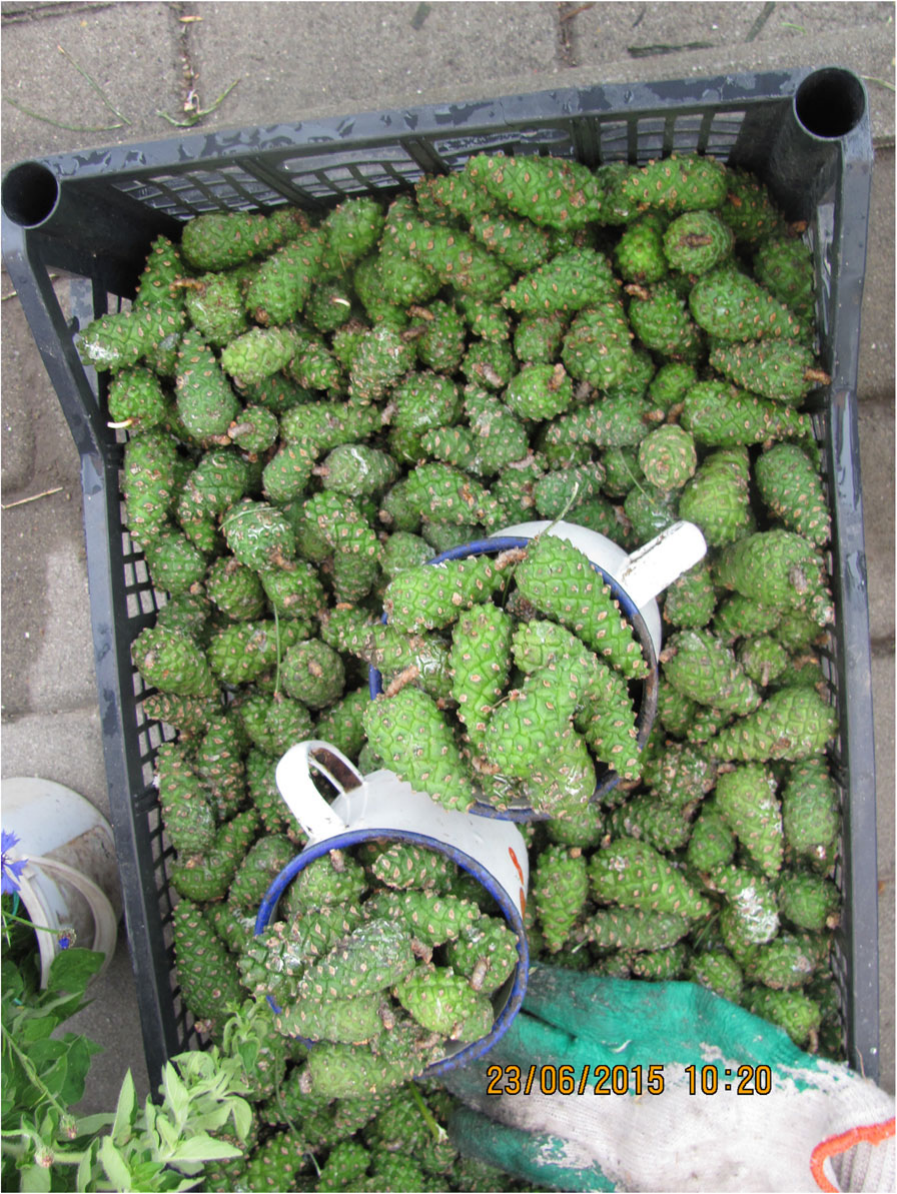
Young pine cones sold for cough syrup in Przemyśl

**Fig. 8:**
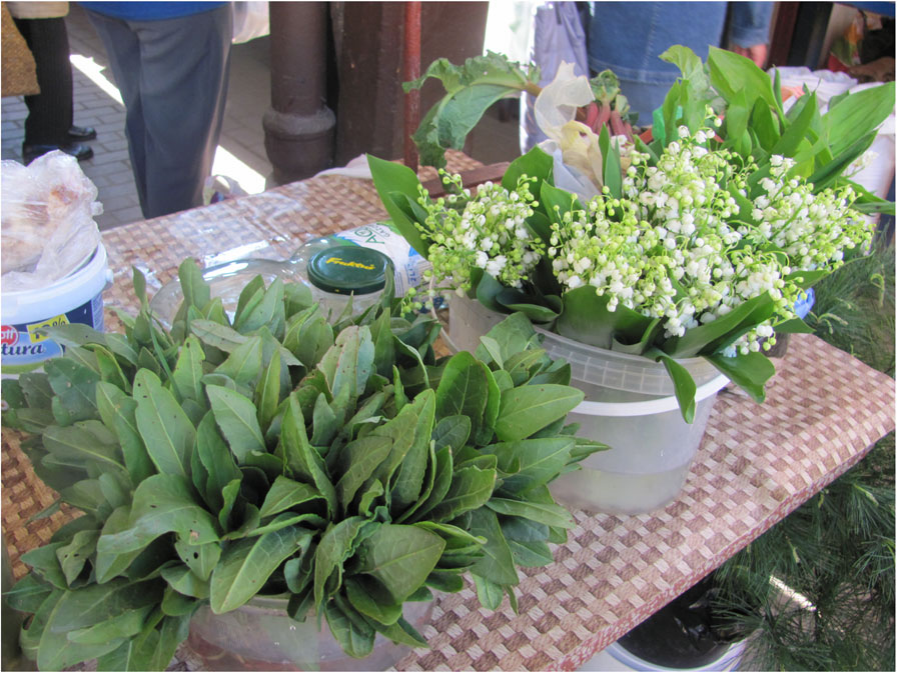
*Convallaria majalis* bouquets and *Rumex acetosa* bunches on sale in Rzeszów

**Fig. 9:**
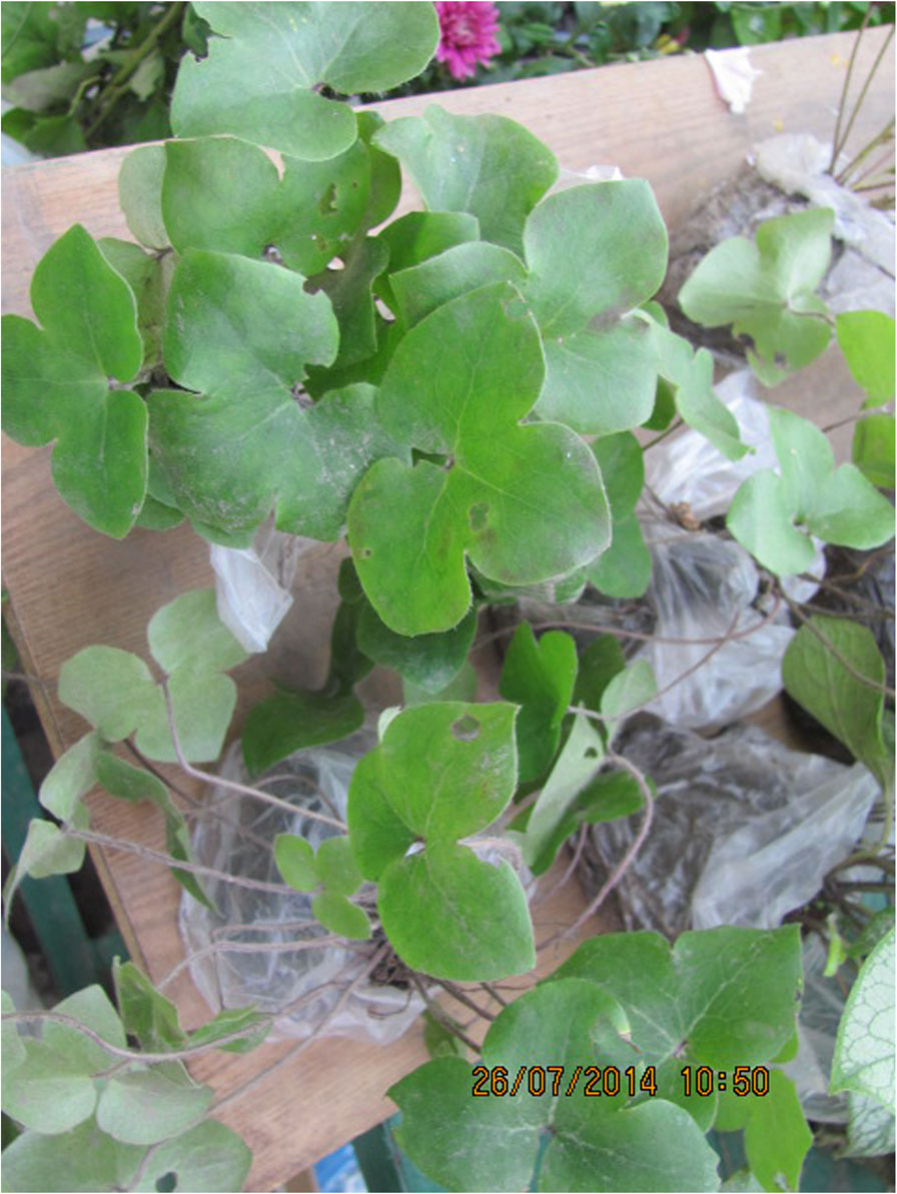
Wild-origin *Hepatica nobilis* sold as a garden ornamental in Rzeszów

Photographs were taken on every visit in order to capture the diversity of cultivated and wild plants sold in the markets (Figs. 2, 3, 4, 5, 6, 7, 8 and 9). Lists of species sold were then made for each visit. Voucher specimens were collected if possible. Unfortunately, usually only parts of plants were available as vouchers and sometimes sellers refused to donate even parts of the plants they sold. The second part of the study consisted of interviews with the plant sellers. Altogether, 100 interviews were conducted (25 in each of the four markets). The interviews were carried out in the markets with the owners of plant stalls. The mean age of sellers was 55 (median 58.5). The oldest seller was 83, the youngest 22. There were 62 women and 38 men in the interviewed group. Around half of the sellers were farmers, the rest – a variety of professionals, often retired. Profit and hobby were equally frequently cited motivations for selling plants in the market. The sellers had been selling plants for an average of 19 years (maximum selling time 50 years). As many as 62 % of sellers came every day, and the remainder 3–4 times a week.

The origins of the species (cultivation versus wild) were established based on interviews and the containers in which the species were sold. For example, species sold in small pots without any weeds and with well-established roots and one shoot were treated as cultivated, whereas species sold in plastic bags, dug out, with traces of natural vegetation (e.g. woodland mosses, other woodland or semi-natural grassland plants), were suspected to be collected from the wild (although they were sometimes dug out from gardens but then there were no traces of forest vegetation). In most cases the answers of the respondents were treated as trustworthy, with the exception of protected or believed-to-be-protected plants - sometimes sellers hesitated or gave very unclear answers when asked if the plant was dug out from the wild. In the case of strange behaviour from informants, we treated the plant as originating from the wild.

Voucher specimens of plants and fungi were deposited in the herbarium of the University of Warsaw (WA). Plants were identified using the standard identification key concerning local floras, and their names follow the Plant List [47]. The status of the plants in the region (native versus non-native established species) was checked with the atlas of the distribution of vascular plants in Poland [48] and other publications on the local flora. Fungi names follow the Index Fungorum [49].

Most fungi specimens were successfully identified using the DNA barcoding technique [50, 51]. Fungal DNA was extracted from a small part of each fruiting body using a Plant and Fungi DNA Purification Kit (Eurx), following standard protocol. The PCR cocktail was composed of 4 ml DNA extract, 0.5 ml each of the primers (ITS5 and ITS4 in 10 nmol concentration) and 5 ml Type-it Microsatellite PCR Kit (Qiagen). PCR was performed using the following thermocycling conditions: an initial 15 min at 95 °C, followed by 35 cycles at 95 °C for 30 s, 55 °C for 30 s, 72 °C for 1 min, and a final cycle of 10 min at 72 °C. PCR products were estimated by running 5 ml DNA amplicon on 1.5 % agarose gel for 30 min. The PCR products were sequenced with the use of ITS4 primers, at the Laboratory of Molecular Biology of Adam Mickiewicz University (Poznań). The obtained sequences were verified visually on chromatograms using BIOEDIT. Nuclear ITS sequences obtained in this study are deposited in GenBank [52], with the accession numbers listed in Table 1.

**Table 1:** The results of DNA barcoding

Molecular identification	Voucher number, starting from WA00000	Accession number	Best match sequence (accession number)	E-value	Similarity (%)
*Agaricus bisporus* (J.E. Lange) Imbach	52304	KX756391	Agaricus bisporus (LK024175)	0.0	99.56
*Armillaria mellea* (Vahl) P. Kumm	52261	KX756392	Armillaria mellea (AM269762)	0.0	98.55
*Armillaria ostoyae* (Romagn.) Herink	52259	KX756393	Armillaria ostoyae (JN657462)	0.0	99.83
*Boletus edulis* L.	52266	KX756394	Boletus edulis (KC750230)	0.0	100
	52295	KX756395	Boletus edulis (KC750230)	0.0	100
	52300	KX756396	Boletus edulis (DQ131623)	0.0	99.86
*Boletus luridiformis* Rostk.	52272	KX756397	Boletus erythropus (UDB001523)	0.0	99.85
*Boletus luridiformis* Rostk.	52289	KX756398	Boletus erythropus (UDB001523)	0.0	99.85
*Chalciporus piperatus* (Bull.) Bataille	52248	KX756399	Chalciporus piperatus (UDB001528)	0.0	98.83
*Cortinarius caperatus* (Pers.) Fr	52277	KX756400	Cortinarius caperatus (KC842443)	0.0	100
*Imleria badia* (Fr.) Vizzini	52246	KX756401	Imleria badia (HM190050)	0.0	99.82
	52247	KX756402	Imleria badia (HM190050)	0.0	98.72
	52249	KX756403	Imleria badia (HM190050)	0.0	99.27
	52250	KX756404	Imleria badia (HM190050)	0.0	100
	52252	KX756405	Imleria badia (HM190050)	0.0	99.82
	52254	KX756406	Imleria badia (HM190050)	0.0	99.36
	52255	KX756407	Imleria badia (HM190050)	0.0	100
	52263	KX756408	Imleria badia (HM190050)	0.0	99.65
*Lactarius quieticolor* Romagn	52283	KX756409	Lactarius quieticolor (UDB001593)	0.0	100
*Lactarius salmonicolor* R. Heim & Leclair	52281	KX756410	Lactarius salmonicolor (DQ679801)	0.0	100
	52296	KX756411	Lactarius salmonicolor (UDB000370)	0.0	100
	52303	KX756412	Lactarius salmonicolor (UDB000370)	0.0	99.27
	52305	KX756413	Lactarius salmonicolor (UDB000370)	0.0	99.71
*Leccinum schistophilum* Bon	52294	KX756414	Leccinum schistophilum (UDB019543)	0.0	99.10
*Leucoagaricus nympharum* (Kalchbr.) Bon	52299	KX756415	Leucoagaricus nympharum (JQ683121)	0.0	100
*Pleurotus cornucopiae* (Paulet) Rolland	52287	KX756416	Pleurotus cornucopiae (KP877606)	0.0	99.53
*Polyporus umbellatus* (Pers.) Fr.	52306	KX756417	Polyporus umbellatus (UDB022812)	0.0	99.65
*Sparassis crispa* (Wulf.) Fr.	52290	KX756418	Sparassis crispa (KC987583)	0.0	98.94
	52307	KX756419	Sparassis crispa (UDB018795)	0.0	99.48
*Suillus bovinus* (L.) Roussel	52265	KX756420	Suillus bovinus (KF482482)	0.0	100
	52271	KX756421	Suillus bovinus (GU016620)	0.0	99.68
	52282	KX756422	Suillus bovinus (GU016620)	0.0	100
	52288	KX756423	Suillus bovinus (GU016620)	0.0	99.68
	52292	KX756424	Suillus bovinus (GU016620)	0.0	100
*Suillus grevillei* (Klotzsch) Singer	52245	KX756425	Suillus grevillei (UDB015555)	0.0	99.93
	52262	KX756426	Suillus grevillei (UDB015666)	0.0	99.93
*Suillus luteus* (L.) Roussel	52270	KX756427	Suillus luteus (JX907818	0.0	99.84
	52274	KX756428	Suillus luteus (JX907818)	0.0	100
	52279	KX756429	Suillus luteus (UDB000930)	0.0	100
	52280	KX756430	Suillus luteus (LC035286)	0.0	100
	52286	KX756431	Suillus luteus (JX907818)	0.0	99.69
	52293	KX756432	Suillus luteus (UDB000930)	0.0	99.69
	52298	KX756433	Suillus luteus (JX907818)	0.0	100
*Suillus variegatus* (Sw.) Kuntze	52291	KX756434	Suillus variegatus (AJ971399)	0.0	100
*Tricholoma equestre* (L.) P. Kumm.	52268	KX756435	Tricholoma equestre (UDB011389)	0.0	100
	52269	KX756436	Tricholoma equestre (UDB011389)	0.0	100
*Tricholoma frondosae* Kalamees & Shchukin	52278	KX756437	Tricholoma frondosae (LT000169)	0.0	100
*Xerocomellus cisalpinus* (Simonini et al.) Klofac	52253	KX756438	Xerocomellus cisalpinus (UDB002180)	0.0	99.53

## Results and discussion

### General information

We recorded 468 species of plants sold in the studied markets, including 117 species of plants which are native to Poland, or aliens (anthropophytes) established in the Podkarpacie region (Table 2). However the actual origins of native plants sold in the markets vary and included cultivated plants (84), plants which are both cultivated and collected from the wild (13 species), and species collected only from the wild (19 species).

**Table 2:** Plants native to Poland and alien plants which have wild populations in the region sold in south-eastern Poland in open-air markets

Latin name - taxa which are exclusively or mainly collected from the wild are written in bold	Voucher specimen no. starting from WA00000	Frequency: 1 – one seller, 2- two sellers, 3 – more than two sellers				Name used in the market	Native status	Form of sale	Origin of sold plants	Purpose of sale
		R	J	L	P					
*Achillea millefolium* L.	52313	1				krwawnik	R	pots	cult. & wild	med.
*Aconitum firmum* Rchb.^a^		1				tojad	P	pots	cult.	orn.
*Acorus gramineus* Sol. ex Aiton	52314	1				tatarak	A	pots	cult.	orn.
*Ajuga reptans* L.	52315	2		2	1	dąbrówka	R	pots	cult.	orn.
*Alchemilla vulgaris* L.	52316	2	1	1		przywrotnik	R	pots	cult.	orn.
*Allium ursinum L.* ^b^	52317	3	3		3	czosnek niedźwiedzi	R	pots, leaves	cult. & wild	food, orn.
*Anemone pulsatilla* L.	52318	3	3	3	2	sasanka	P	pots	cult.	orn.
*Anemone sylvestris* L.^b^	52319	1	1	2		zawilec leśny, zawilec biały	R	pots	cult.	orn.
*Anemone vernalis* L.^b^		3	3	2	2	sasanka	P	pots	cult.	orn.
*Arabis alpina* L.	52309	3	1	2	1	gęsiówka	P	pots	cult.	orn.
*Arenaria serpyllifolia* L.	52320	2		1	1	piaskowiec	P	pots	cult.	orn.
*Armeria maritima* Willd.	52321	3	3	3		zawciąg	R	pots	cult.	orn.
*Armoracia rusticana* G. Gaertn.		3	3	3	3	chrzan	A	roots	cult. & wild	food (spice)
*Artemisia absinthium* L.	52322	3	1	1		piołun	R	bare rooted plants	cult. & wild	med.
*Aruncus dioicus* (Walter) Fernald	52323			1			R	pots	cult.	orn.
*Asarum europaeum* L.	52324	1		1	1	kopytnik	r	pots	cult.	orn.
*Asparagus officinalis* L.	52325	1				szparag	r	bare rooted plants	cult.	food
*Astrantia major* L.	52326			1		jarzmianka	r	pots	cult.	orn.
*Bellis perennis* L.	52327	3	2	2	1	stokrotka	r	bouquets, pots	cult.	orn.
*Berberis vulgaris* L.	52328			1		berberys	r	fruits	cult.	food
*Calluna vulgaris* (L.) Hull	52329	3	3	3	2	wrzos	r	pots	cult. andwild	orn.
*Caltha palustris L.*	52330	1			1	kaczyniec, kaczeniec	r	pots	wild	orn.
*Campanula glomerata* L.		1	1	2		dzwonek	r	pots	cult.	orn.
*Campanula persicifolia* L.		2		2	1	dzwonek	r	pots	cult.	orn.
*Cardamine glandulifera* O.Schwarz		1				-	r	bare rooted plants	cult.	orn.
*Carlina acaulis* L.^b^	52331	1		1		dziewięćsił	r	pots	cult.	orn.
*Carum carvi* L.	52332	1				kminek	r	pots	cult.	spice
*Centaurea scabiosa* L.						-	r	pots	cult.	orn.
*Convallaria majalis* L.	52333	3	3	2	3	konwalia	r	pots, bouquets	cult. & wild	orn.
*Corylus avellana* L.		3	3	3	3	orzech laskowy	r	fruits	cult. & wild	food
*Crataegus* spp.		2	1		1	głóg	r	fruits	wild	food, alc., med.
*Crocus vernus* (L.) Hill (including *Crocus scepusiensis* (Rehm. et Woł. ^b^)		*2*				krokus fioletowy	p	pots	cult.	orn.
*Cyanus montanus* (L.) Hill.	52334		1			chaber	r	pots	cult.	orn.
*Cyanus segetum* Hill.	52335	2	3	2	1	bławatek	a	bouquets, wreaths	wild	orn.
*Cytisus scoparius* (L.) Link	52336	2	1			żarnowiec (żółty)	r	pots	cult.	orn.
*Daucus carota* L.		*3*	*3*	*3*	*3*	marchew	r	roots	cult.	food
*Delphinium elatum* L.	52337	3	2	3	2	ostróżka	r	pots	cult.	orn.
*Dianthus carthusianorum* L.		1			1	kartuzek	r	pots	cult.	orn.
*Dianthus plumarius* L.	52338	3	1	2	1	pierzasty ochr.	p	pots	cult.	orn.
*Dictamnus albus* L. ^a^	52339	1	1	1		gorejący krzew Mojżesza	p	pots	cult.	orn.
*Digitalis grandiflora* Mill.^b^	52340	1	2	1		naparstnica	r	pots	cult.	orn.
*Digitalis purpurea* L.		3	1	2	1	naparstnica	a	pots	cult.	orn.
*Dryas octopetala* L.	52341			1		dębik	p	pots	cult.	orn.
Echinops exaltatus Schrad.		2			1	przegorzan	a	pots	cult.	orn.
*Echinops sphaerocephalus* L.	52342	1			1	przegorzan	a	doniczki	cult.	orn.
*Eryngium planum* L.	52375	1				mikołajek	r	bukiety	cult.	orn.
*Euphorbia amygdaloides* L.	52343	3	2	2		wilczomlecz	r	pots	cult./wild.	orn.
*Filipendula vulgaris* Moench	52344	1		1		wiązówka	r	pots	cult.	orn.
*Fragaria vesca* L.		3	3	2	2	poziomka	r	fruits	cult.	food
*Galanthus nivalis* L.^b^	52345	1				śnieżyczka	r	bouquet	cult.	orn.
*Gypsophila paniculata* L.^b^	52346			1	2	gipsówka bukietowa	p	pots	cult.	orn.
*Helianthus tuberosus L.*		*1*				topinambur	a	tubers	cult.	food, ornamental
*Hepatica nobilis* Mill.	52347	2	1			przylaszczka	r	pots	cult. and wild	orn.
*Hypericum perforatum* L.	52348	1	1		1	dziurawiec	r	dried aerial parts	wild	med.
*Inula helenium* L.			1	1		oman	a	pots	cult.	orn.
*Iris pseudacorus* L.	52349	3	3	3	2	irys	r	pots	cult.	orn.
*Iris sibirica* L.	52350	3	2	1	2	irys fioletowy	r	pots	cult.	orn.
*Juniperus communis* L.				1		jałowiec	r	pots	cult.	orn.
*Lamiastrum galeobdolon* (L.)L.		1				gajowiec	r	pots	cult.	orn.
*Ledum palustre* L. ^b^	52351	3	3	1		bagno	r	aerial parts	wild	ins.
*Leontopodium nivale* (Ten.) Huet ex Hand.-Mazz.^a^	52352	2	1	1		szarotka	r	pots	cult.	orn.
*Leucanthemum vulgare* (Vaill.) Lam.	52353	3	3	3	3	margaretka/margerytka	r	bukiety,	cult.	orn.
*Leymus arenarius* (L.) Hochst.				1		nadmorska	p	pots	cult.	orn.
*Ligularia sibirica* (L.) Cass.^a^				1		języczka	p	pots	cult.	orn.
*Lupinus polyphyllus* L.		3	3	2		łubin	a	pots	cult.	orn.
*Lysimachia punctata* L.	52354		1	1		tojeść	a	pots	cult.	orn.
*Matricaria chamomilla* L.	52355	2	1	1		rumianek	a	inflorescences	cult.	med.
*Matteucia struthiopteris* (L.)Tod.	52356				1	paprotka	r	pots	cult.	orn.
*Myosotis sylvatica* Hoffm.	52357	2		1		niezapominajka	r	pots	cult.	orn.
*Oenothera* sp.		1				wiesiołek	a	pots	cult.	orn.
*Origanum vulgare* L.	52358	3	1	1	1	oregano	r	pots	cult. & wild	spice
*Ornithogalum candicans* (Baker) J.C.Manning & Goldblatt				1		galtonia	r	pots	cult.	orn.
*Ornithogalum umbellatum* L.^a^	52310	3	1		2	śpioch do 2014	r	pots	cult.	orn.
*Pinus sylvestris* L.		1	2		1	sosna	r	young shoots	wild	alc., med.
*Polemonium coeruleum* L.					1	-	p	pots	cult.	orn.
*Polygonatum multiflorum* (L.) All.					1	kokoryczka	r	pots	wild	orn.
*Primula elatior* (L.) Hill^b^		2	2	1		pierwiosnek	r	pots	cult.	orn.
*Primula veris* L.			1			pierwiosnek	r	pots	cult.	orn.
*Primula vulgaris* Huds.	52359	3	3	3	2	pierwiosnek	r	pots	cult.	orn.
*Prunus avium* L.		3	3	3	3	czereśnia	r	fruits	cult.	food
*Prunus spinosa* L.		2				tarnina	r	fruits	wild	alc.
*Pulmonaria officinalis* L. s.l.	52360	2	1	2		miodunka	r	pots	cult.	orn.
*Pyrus communis* L.		3	3	3	3	gruszka	r	fruits	cult.	food
*Ribes nigrum* L.		3	3	2	2	porzeczka	r	fruits	cult.	food, alc.
*Ribes uva-crispa* L.		2	2	1	2	agrest	r	fruits	cult.	food
*Rosa canina* L.	52361	2	2		2	róża	r	fruits	wild	food, alc., med.
*Rubus idaeus* L.		3	3	3	3	malina	r	fruits	cult.	food
*Rubus subgenus Rubus*		3	2		1	ostrężyna	r	fruits	wild	food
*Rumex acetosa* L.	52362	3	3		3	szczaw	r	bundles of leaves	cult. & wild	food
*Rumex sanguineus* L.				1	1	-	r	pots	cult.	food
*Salix caprea* L.	52363	3	3	2		bazie	r	leafless twigs with catkins	wild	cer. for Easter
*Salix* cf *purpurea* L.			1	1		wiklina	r	leafless twigs with catkins, craft material	cult.	cer. for Easter
*Salvia nemorosa* L.	52311	2	1	1		szałwia omszona	p	pots	cult.	orn.
*Sambucus nigra* L.		1				czarny bez	r	racemes of fruits	wild	food, alc., med.
*Sedum acre* L.	52364	2	1	1		rozchodnik	r	pots	cult.	orn.
*Sempervivum globiferum L.*		1				rojownik	r	pots	cult.	orn.
*Silene viscaria* (L.) Jess.	52365		1	2	1	smółka	r	pots	cult.	orn.
*Sorbus aucuparia* L.		2			1	jarzębina	r	fruits	wild	food, alc., med.
*Staphylea pinnata* L.		1				kłokoczka	r	pots	cult.	orn., religious (making rosaries)
*Tanacetum parthenium* (L.) Sch. Bip.	52366	1		1		maruna	a	bouquet	cult.	orn.
*Tanacetum vulgare* L.	52367	1				wrotycz	r	pots, bundles	wild	med.
*Thalictrum aquilegifolium* L.				1		rutewka	r	pots	cult.	orn.
*Thymus serpyllum* L.	52368	2	1	1	1		r	pots	cult.	orn.
*Tilia cordata* Mill.	52369	1				lipa	r	inflorescences	wild	med.
*Tripleurospermum maritimum* (L.) W. D. J. Koch			1	1			a	pots	cult.	orn.
*Trollius europaeus* L.^a^		2	2			pełnik	r	pots	cult.	orn.
*Vaccinium myrtillus* L.		3	3	3	3	czarna jagoda	r	fruits	wild	food, med.
*Vaccinium vitis-idaea* L.		2	2	1	2	brusznica	r	fruits	wild	food
*Valeriana officinalis* L.	52370		1			kozłek	r	pots	cult.	med.
*Veronica spicata* L.	52371	3		1	1	przetacznik	r	pots	cult.	orn.
*Viburnum lantana* L.			1			kalina	p	pots	wild	orn.
*Viburnum opulus* L.	52372	1	1		1	kalina	r	fruits	wild	food, med.
*Vinca minor* L.	52373	1		1	1	barwinek	r	pots	cult.	orn.
*Viola alba* Besser^b^		2	1	1		fiołek	p	pots	cult.	orn.
*Viola odorata* L.	52312	2	1		1	fiołek	a	pots	cult. & wild	orn.
*Viola riviniana* Rchb.	52374				1	fiołek	p	pots	cult.	orn.
*Viscum album* L.				1		jemioła	r	aerial parts	wild	cer. for Christmas

*R* Rzeszów, *J* Jarosław, *L* Leżajsk, *P* Przemyśl

*r* native to the region, *p* native to Poland but not occurring in the region, *a* anthropohyte

*cult.* cultivated, *orn.* ornamental, *alc.* alcoholic drinks, *med.* medicinal, *ins.* insect repellent, *cer.* ceremonial

^a^fully protected species, ^b^partially protected species

**Fig. 10:**
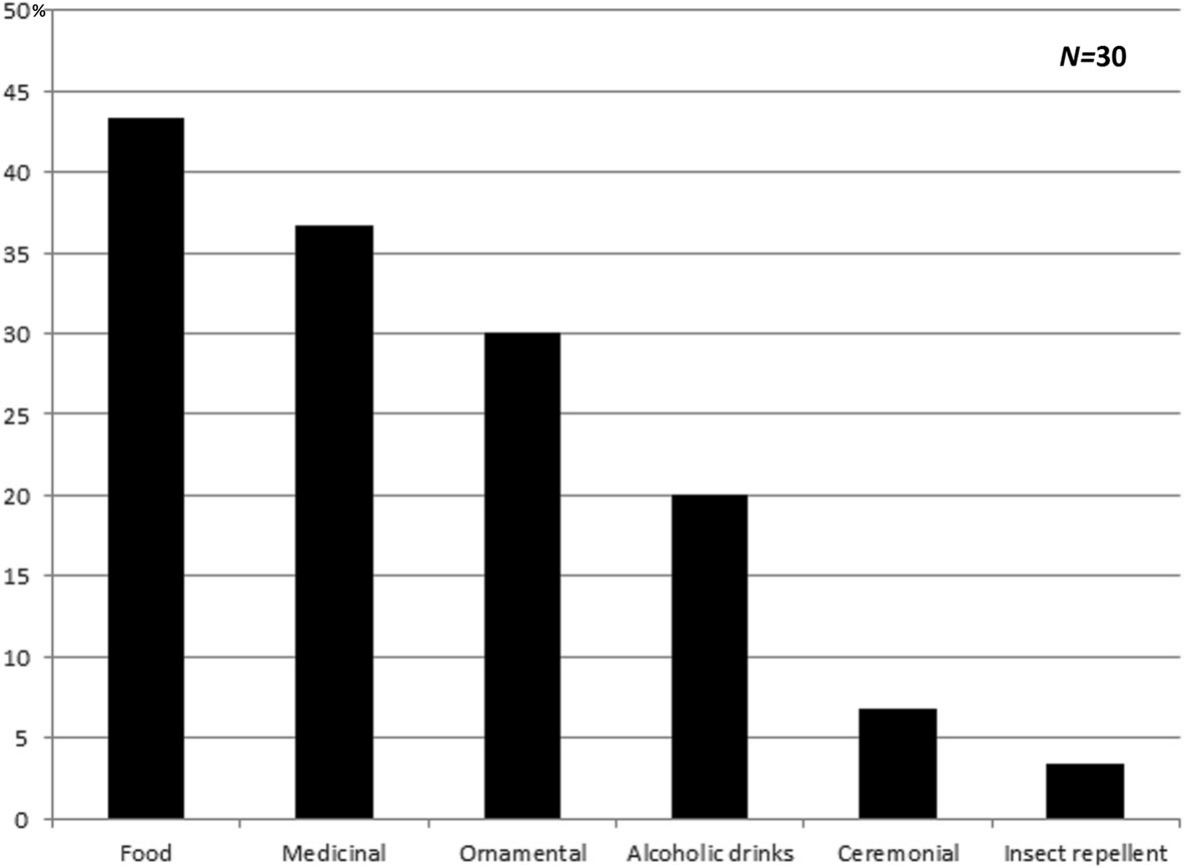
Use categories for species collected from the wild

**Fig. 11:**
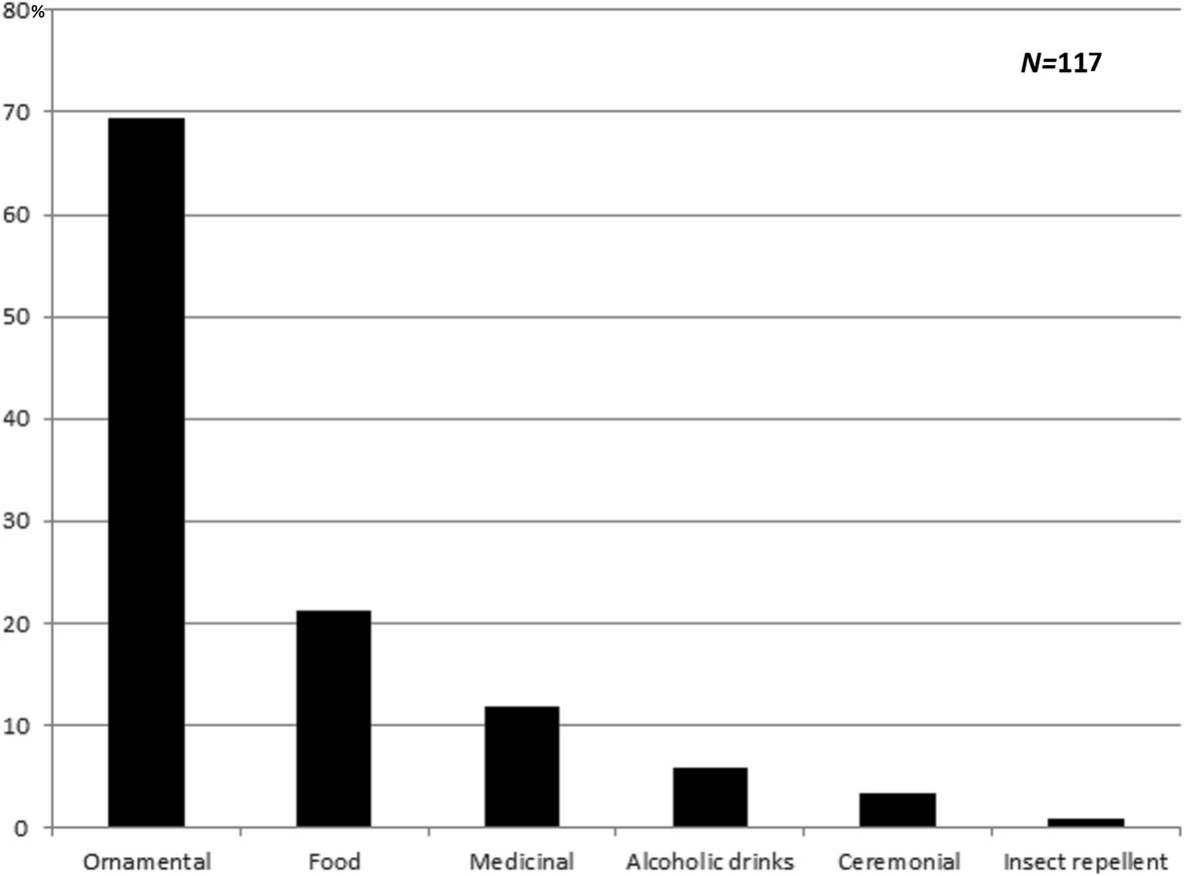
Use categories for all species which could potentially occur in the wild (native and naturalized)

When only the plants which are at least partly collected from the wild are taken into account, most are sold for food, more rarely for medicinal and ornamental purposes (Fig. 10). When cultivated native plants are added, the ornamental purpose becomes dominant (Fig. 11), followed by food use and medicinal use. There are very few medicinal plants sold solely for this purpose (e.g. *Tanacetum vulgae*, *Hypericum perforatum*). Most medicinal plants are fruits used both for food and alcoholic drinks, and as medicine.

As many as 32 species of fungi are sold, all of them for culinary purposes (Table 3), 30 of them are collected from the wild.

**Table 3:** Edible mushrooms sold in the markets of south-eastern Poland

Latin name	Name used in the market	Voucher specimen number starting from WA00000	Frequency: 1 – one seller, 2- two sellers, 3 – more than two sellers			
			Rzeszów	Jarosław	Leżajsk	Przemyśl
*Agaricus bisporus* (J.E. Lange) Imbach^CULT^	pieczarka	52304	3	2		
*Agaricus campestris* L.	pieczarka	-	1			
*Armillaria gallica* Marxm. & Romagn^#^	opieńka	52284, 52308	1			
*Armillaria mellea* (Vahl) P. Kumm[	opieńka	52261	2	1		1
*Armillaria ostoyae* (Romagn.) Herink^#^	opieńka	52259	3	2		2
*Boletus edulis* L.	prawdziwek	52266, 52295, 52300	3	3	3	3
*Boletus impolitus* Fr.^#^		52256		1		
*Boletus luridiformis* Rostk.^#^	podciecz, borowik ceglastopory	52272, 52276, 52289	2	1	1	1
*Boletus subtomentosus* L.	zajączek	52258	2	3		1
*Cantharellus cibarius* Fr.	kurka	52302	3	3	3	3
*Chalciporus piperatus* (Bull.) Bataille^1#^	maślak	52248	1			
*Cortinarius caperatus* (Pers.) Fr	kołpak, chochołka	52277			1	
*Imleria badia* (Fr.) Vizzini	poddąbek, podgrzybek	52246, 52247, 52249, 52250, 52254, 52255, 52263	3	3	3	3
*Lactarius deliciosus* (L.) Gray	rydz	52275	*3*	3	2	1
*Lactarius quieticolor* Romagn.^2#^	rydz	52283			1	
*Lactarius salmonicolor* R. Heim & Leclair	rydz	52281, 52296, 52303, 52305	*3*	2	1	1
*Leccinum aurantiacum* (Bull.) Gray	czerwony kozak	52271, 52285, 52288, 52297	*3*	3	1	2
*Leccinum scabrum* (Bull.) Gray	kozak	52264, 52273, 52301	3	2	1	2
*Leccinum schistophilum* Bon^3#^	kozak	52294	1			
*Leucoagaricus nympharum* (Kalchbr.) Bon^4#^	kania	52299	1			
*Macrolepiota procera* (Scop) Singer	kania	52251	3	3	1	2
*Pleurotus cornucopiae* (Paulet) Rolland^#CULT^	boczniak	52287	1			
*Polyporus umbellatus* (Pers.) Fr.^a#^	żagiew	52306	1			
*Sparassis crispa* (Wulf.) Fr.^#^	szmaciak	52290, 52307		2		1
*Suillus bovinus* (L.) Roussel	maślak sitarz	5265, 5282	3	2	1	1
*Suillus granulatus* (L.) Roussel	maślak	52265, 52282, 52292	1	1		1
*Suillus grevillei* (Klotzsch) Singer	maślak modrzewiowy	52245, 52262	3	3		2
*Suillus luteus* (L.) Roussel	maślak	52270, 52274, 52279, 52280, 52286, 52293, 52298	3	3	2	3
*Suillus variegatus* (Sw.) Kuntze	bagniak	52267, 52291	2	1	1	
*Tricholoma equestre* (L.) P. Kumm.	gąska	52268, 52269	3	1	1	
*Tricholoma frondosae* Kalamees & Shchukin^#^	gąska	52278	1			
*Xerocomellus cisalpinus* ^#^ (Simonini et al.) Klofac	podgrzybek	52253				1

^a^protected species

#species not present in the official list of mushroom species allowed for sale

^CULT^cultivated species

^1^It has peppery taste, confused with *Suillus* spp

^2^Confused with *Lactarius deliciosus* or *L. salmonicolor*, probably an accidental admixture

^3^Confused with *Leccinum scabrum*, probably an accidental admixture

^4^Confused with *Macrolepiota*, probably an accidental admixture

Rzeszów is the largest market and hosts the highest biodiversity: 91 species of native or established alien species, compared to 67 in Jarosław, 72 in Leżajsk and 55 in Przemyśl. The largest number of wild-origin species are also sold in Rzeszów (27), compared to 22 in Jarosław, 20 in Przemyśl and 13 in Leżajsk. The largest number of fungi are also sold there (26), compared to 21 in Jarosław, 18 in Przemyśl and 15 in Leżajsk (Table 3).

The interviewed sellers mentioned 46 taxa of plants which they thought had been sold more often in the past, but they mentioned over twice as many (103 taxa, including non-native cultivated plants) as novelties, only recently sold. Out of the species which are sold less, the only wild/native species was nettle *Urtica dioica*, mentioned by 2 informants. From the species which are seen as new or increasing, the informants mentioned a few native plants. Predominantly mentioned were ramsons *Allium ursinum* (5 informants), wormwood *Artemisia absinthium* (4), sorrel *Rumex acetosa* (3), *Delphinium*, blueberry *Vaccinium* spp. (2), sea buckthorn *Hippophae rhamnoides* and edelweiss *Leontopodium alpinum* (Table 4).

**Table 4:** “Protected” plants and fungi (in the emic sense) sold in open-air markets according to sellers – regardless of their real protection status. The table includes those mentioned at least twice

Scientific name	Local name	No. of informants	Biogeographical status and conservation status	Origin
*Allium ursinum* L.	czosnek niedźwiedzi	11	locally abundant in the region partly protected	mainly collected from the wild, sold in pots and leaves used as a vegetable
*Convallaria majalis* L.	konwalie	7	locally abundant in the region protected until 2014	both from gardens and forests, as cut flowers
*Lycopodium* spp.	widłak	6	locally frequent partly protected	from the wild, not observed on sale
*Pulsatilla* spp.	sasanka	5	practically extinct protected	sold by specialist sellers, from cultivation
*Galanthus nivalis* L.	przebiśnieg	3	abundant but only very locally partly protected	mainly from cultivation
Orchidaceae	storczyk	3	locally abundant protected	not observed on sale
*Leontoodium alpinum* L.	szarotka	3	not native to the region protected	from cultivation
*Carlina acaulis* L.	dziewięćsił	3	rare partly protected	from cultivation
*Hepatica nobilis* L.	przylaszczka	2	locally frequent, protected until 2014	from cultivation and from the wild
*Polygonatum multifolorum* L.	kokoryczka	2	frequent, not protected not protected	from cultivation and from the wild
*Daphne mezereum* L.	wawrzynek	2	occasional partly protected	from cultivation and from the wild
*Sparassis crispa* (Wulfen) Fr.	baraniocha	2	frequent, protected until 2014	from the wild

### Protected plants

Relatively few protected plants (i.e. protected in the emic sense, according to respondents’ knowledge) were listed as sold. Only 12 taxa of protected plants were mentioned by more than one informant. *Allium ursinum*, *Convallaria maialis* and *Lycopodium* were the most often cited. Most of the protected plants were cultivated in gardens (Table 1). This is especially the case with highland plants native only to the highest parts of the Carpathians (*Leontopodium alpinum*, *Dryas octopetala* etc.). The species which may be taken from the wild are *Allium ursinum*, club-mosses and orchids. (Un)fortunately club-mosses were not seen on sale in the study period. The sellers of ramsons (*Allium ursinum*) claim they are cultivated but they have no certificate from local nature conservation authorities, and we suspect that the plants come from wild populations.

Most of the protected plants are sold due to their ornamental value (apart from ramsons, whose use as vegetable has recently become fashionable). Poland is not the only country where plants with beautiful flowers are endangered. For example in Mexico the trade of ornamental orchids creates a biodiversity problem [34].

A very interesting issue is the difference between ordinary people’s perceptions of protected plants and which plants are really protected. Our observations allow us to hypothesize that people are over-cautious about plant protection. There are some species which they think are protected but have never been, e.g. *Anemone nemorosa*. This wild species is common in the woods in Poland and many people think that it is protected by law because they learned at school about the protection of *Anemone sylvestris* and *A. narcissiflora*. Recently, in 2014, protection status was removed from many commoner species, which had previously been protected to avoid their being overharvested as medicinal plants (e.g. *Frangula alnus*, *Viburnum opulus*, *Asarum europaeum*, *Convallaria majalis*), or because we now know that they are more common than previously thought (e.g. *Equisetum telmateia*).

### Edible plants and fungi

Wild fruits make up an important sector of plants sold in the markets. From the interviews we infer that some species are re-appearing after years of neglect. The main reason for this re-emergence is the growing popularity of herbal medicine and fruit liqueurs. For example such plants as *Rosa canina*, *Crataegus* or *Prunus spinosa* are mainly sold for alcohol production, to a lesser extent also for herbal teas. This trend of the increasing availability of rarer economic plants in Polish market was already noticed by other authors [7, 53].

Mushrooms constitute another important sector of the open-air markets. The number of recorded mushroom species is relatively high. It must be emphasized that the DNA bar-coding we used enabled confirmation of the identification of some surprising taxa sold in the markets (*Lactarius quieticolor*, *Leccinum schistophilum*, *Leucoagaricus nympharum*) never recorded as food in Poland before. Moreover, *L. quieticolor* and *L. schistophilum* are not listed in the checklist of Basidiomycetes found in Poland [54] and are new to the mycobiota of Poland. This highlights the importance of DNA barcoding in ethnomycological studies, illustrated well by the study of Dentinger and Suz [55] who found threw new species of porcini (*Boletus*) in a single packet of mushrooms sold in London and imported from China.

The list of fungi sold in the markets is similar to the taxa reported from a few ethnographic and ethnomycological studies in south-eastern Poland [56]. On the other hand some mushroom taxa widely collected in rural areas are very rarely sold, for example the *Russula* genus. Russulas can be confused with death cap *Amanita phalloides*, which is why they are not on the list of taxa permitted for sale in markets [37, 57]. On the other hand *Boletus luridiformis*, which is not on the list of taxa allowed for commerce, is more frequently sold (and also traditionally collected here). Altogether 13 species of 32 sold in the markets are not on the list of species legally permitted for sale [57], and even one protected species [58] is sold (Table 3). In some cases very closely related taxa are listed in this legal document (*Armillaria*, *Leccinum*), in others the whole genus is not mentioned (even taking into account taxonomic changes and synonyms).

We may conclude that the choice of mushrooms in markets may be a good indicator of culturally salient edible mushrooms, though some lesser collected taxa may not be visible. A similar relationship was found between the traditionally collected wild vegetables in Dalmatia and those which are sold in the markets of Dalmatian towns [59], where there is high correlation between the two lists of taxa but some differences occur.

### Comparison with other countries

It is difficult to compare Polish open-air markets with other European countries due to the scarcity of available data, which is restricted to south-eastern Europe. It seems that they have different features from the markets studied in Croatia, Bulgaria and the European part of Turkey. Turkish and Croatian markets sell a large number of wild green vegetables [10–14]. In Poland these are mainly restricted to *Rumex* species. Bulgarian markets, similarly to the Polish ones, have few wild vegetables, but contain many medicinal plants [13, 14]. Such plants used to be sold in Polish markets in the first half of the 20th century [3–6]. Nowadays purely medicinal plants are rare in Polish markets but the category of culinary herbs is fashionable, however they are mainly non-natives species. Such species as basil or oregano are relatively new to mainstream Polish cuisine and became fashionable a few years ago. People may be scared to sell purely medicinal plants due to regulations concerning the sales of medicinal material.

## Conclusions

The open-air markets of southeastern Poland sell a considerable number of native plants but only a small proportion of them come from wild populations (mainly edible fruits). Most items are ornamental plants, or edible fruits and mushrooms. Very few medicinal plants and green vegetables are sold, which differentiates the markets from southern European ones. Such a pattern is probably the model for most central European markets, but no similar research has been carried out in neighbouring countries.

Finding two species of fungi which are new to Poland highlights the importance of DNA barcoding in ethnomycological studies.
